# The Ward Wall

**Published:** 1911-09-23

**Authors:** 


### Hospital Architecture and Construction.

[Communications on this subject should be marked "Architecture" in the left-hand top corner of the envelope.]

### THE WARD WALL.

In a previous series of articles the finish of the ward floor was fully discussed; the conclusion arrived at was that the best type of material for a ward floor is a composition of asbestos, sawdust, etc., with a lime matrix, coloured with mineral oxide. This composition will finish against the wall with a cavetto or rounded skirting for the wall finish to stop on and to avoid dust-holding angles.

The nature of this material is such that it can be made in many colours and is applied more or less in the same manner as cement. It can be applied as a wall finish, giving an open field for any manner of decoration; but owing to its composition it has a tendency to bulge from the wall unless great care is taken in the setting, and its cost is considerably greater than other materials which give an equally good result.

Glazed bricks have in past years been used for ward wall linings, and have the advantage that there is no discoloration from damp; efflorescence when present can be easily cleaned off, but even with the best quality of work and material it is almost impossible to avoid uneven joints which may harbour dust. The same objection applies to tile linings, though in a lesser degree; but they have the advantage of lending themselves to a more cheerful colour scheme than glazed bricks. The cost, however, is against wall tiling also, as the cheapest work costs about 8s. a yard. The condensation of moisture on such surfaces as brick or tiles is a serious drawback to their use.

Portland cement and Keene's cement are still greatly in favour as a wall finish; the brickwork should be as dry as possible before they are applied, else stains will come through. These cements now have keen competitors in the several new plasters which have recently come forward, and one of them we believe is specified for use in the new King's College Hospital. They compare favourably in price with Portland or Keene's cement, are hard and non-absorbent, and further have the desirable quality of setting and drying very quickly.

One firm who manufactures this type of plaster makes a specialty of partitions on an expanded metal core so that it can be used throughout in the whole building on ceilings, walls, and to form the necessary divisions. The Admiralty is using it on the Naval Hospital Works at Shotley Point and Osborne.

The surfaces being plastered and the moisture gone therefrom, the architect or his client must determine the scheme of decoration. Much is to be said in favour of a good quality varnished paper, which will last some ten or fifteen years if varnished with the best white copal varnish: the surface presented is hard, dry, non-absorbent, and practically impervious.

Many authorities favour painting on the plaster with some of the excellent paints and enamels now on the market, such as Rystolite, Ripolin, etc. Painting gives scope for the individual ingenuity of the architect, and should be four-coat work, with two coats of varnish or one of enamel. With painted work damp stains will show themselves more readily than through a varnished paper, but among recent developments of wall finishes, water paints, such as Duresco, stand pre-eminent. They may show discoloration at first, but as the wall dries go back to their original hue if care is taken to apply them according to the makers' instructions. Duresco and such water paints give an effective finish which is not quite so metallic as a varnished paper or lead-painted surfaces, with the advantage that condensation is not so great. We would appeal for more consideration by authorities and their architects for real mural decoration where circumstances will permit. There are many ways of approaching this treatment which have not yet been exploited, and fresco painting can be carried out to give an effective appearance within reasonable cost if treated in a broad and simple manner.

